# Road safety attitude and behaviour among motorcycle riders in Ghana: A focus on traffic locus of control and health belief

**DOI:** 10.1371/journal.pone.0309117

**Published:** 2024-08-23

**Authors:** Abdul-Raheem Mohammed, Buhari Gunu Yussif, Mustapha Alhassan

**Affiliations:** 1 Department of Social and Behavioural Change, School of Public Health, University for Development Studies, Tamale, Ghana; 2 Department of Global and International Health, School of Public Health, University for Development Studies, Tamale, Ghana; Tsinghua University, CHINA

## Abstract

Road traffic accident is a leading cause of death and various life deformities worldwide. This burden is even higher among motorcycle riders in lower-to-middle-income countries. Despite the various interventions made to address the menace, the fatalities continue to be on the ascendency. One major area that has received little attention is the attitude and behaviour of motorcycle riders. The present study aimed to examine the contribution of traffic Locus of Control (LoC) and health belief on road safety attitude and behaviour. 317 motorcycle riders participated in the study. The participants completed a questionnaire comprising various sections such as motorcycle riding behaviour, road safety attitude, risk perception, the intention to use helmets, and traffic LoC. The results showed a significant positive correlation between road safety attitude and behaviour (*r* (295) = .33, *p* < .001). Drifting towards internal LoC was associated with more positive behaviour on the roads (*r* (295) = -.23, *p* < .001). Intention to use helmet, health motivation, perceived susceptibility, perceived benefits, and perceived barriers were the factors in the health belief model that were associated with road safety attitude (*r* (295) = .404, *p* < .001). Finally, the multiple linear regression model showed that road safety attitude and traffic LoC made significant contributions to road user behaviour [*F*(3, 293) = 13.73, *p* < .001]. These findings have important implications towards shaping responsible behaviour among motorcycle riders.

## Introduction

Countless lives are tragically lost each year due to road traffic accidents, prompting deep concern over this problem. According to data gathered by the World Health Organization (WHO) in 2018, road accidents are responsible for claiming approximately 1.35 million lives globally [1]. This alarming statistic underscores the global significance of road traffic fatalities, making them the primary cause of death for individuals aged 5 to 29. Moreover, these incidents rank as the eighth leading cause of death across all age groups, surpassing the toll of diseases like tuberculosis, HIV/AIDS, and diarrheal illnesses [1]. The economic ramifications are equally significant, accounting for an estimated 3% of the GDP in most countries. In terms of vulnerability, motorcycle and tricycle riders face heightened risks due to their limited protection compared to occupants of enclosed vehicles [2, 3]. Motorcycle riders are also overrepresented in road fatalities in low- and middle-income countries [4, 5].

Despite efforts to tackle the root causes of these accidents, a critical factor remains overlooked, particularly among road users in lower- and middle-income countries: behaviour [6–9]. While various measures have been implemented to combat this issue, such as law enforcement, infrastructure improvements, and education [10], there is a dearth of evidence concerning the role of attitudes toward road safety behaviour. Attitude, in this context, refers to the acquired inclination to perceive an event, object, or outcome as favourable or unfavourable, encompassing cognitive, affective, and behavioural aspects [11, 12]. The concept of attitudes toward road safety implies the cognitive, affective, and behavioural tendencies that shape our evaluation of actions on the road as acceptable or unacceptable. Consequently, road user behaviours are profoundly influenced by these attitudes. This assertion finds support in numerous studies [11, 13–16], with McIlroy et al. [15] noting a stronger relationship in China compared to Kenya. Although a majority of the studies on attitudes towards road safety focused on drivers [13, 17–20], few studies examined the attitude of motorcycle riders (e.g., [21, 22]). However, this is insufficient considering the settings where those studies were carried out. In lower-to-middle-income countries, motorcycles are a much more popular means of transport compared to cars and other public transport systems and are often significantly represented in road carnages.

Among the factors that are believed to predict attitudes towards road safety and behaviour include the intention to engage in road safety behaviour and traffic Locus of Control (LoC). The intention to engage in acceptable road safety behaviour could be explained by the Health Belief Model [23–25]. According to this model, an individual’s intention to take a course of action such as wearing a protective helmet when riding a motorcycle is determined by key factors such as perceived benefits (i.e., the advantages of engaging in the health-promoting behaviour), perceived severity (i.e., how life-threatening it is by not engaging in the health-promoting behaviour), perceived susceptibility (i.e., the vulnerability of the person in getting sick or dying by not engaging in the health-promoting behaviour), perceived barriers (i.e., the challenges that are likely to impede the health-promoting behaviour), cues to action (i.e., the availability of reminders to engage in health-promoting behaviour), and self-efficacy (i.e., the confidence one has about the health-promoting behaviour) [26, 27]. The Health Belief Model since its inception has become popular in public health literature, especially in health-related behaviour interventions [24, 25]. Some studies have attempted to examine how the application of the health belief model could be associated with the behaviour of drivers and other road users in the occurrence of road traffic accidents [28–31]. Previous studies on the association between intention to engage in a health-promoting behaviour and attitudes towards road safety and behaviour are largely positive [32–34]. Specifically, among the Health Belief Model components, perceived benefits and cues to action are positively associated with safe driving behaviours, just as perceived barriers were negatively associated with positive driving behaviours [28, 35, 36]. Another study found only perceived benefits and perceived barriers predicting seat belt use among drivers in Iran [37]. The trend among bus passengers showed that perceived barriers and perceived severity were predictors of seatbelt use [38].

An important variable that is thought to influence attitudes towards road safety and behaviour is traffic LoC. The term "locus of control" (LoC) refers to an enduring belief about the origin of the cause of or the degree of control over one’s conduct and is categorized into internal and external locus of control [39, 40]. Individuals highly inclined to internal locus of control perceive events to be exclusively under their own control. Persons with a high external locus of control are more inclined to place blame for their acts on other people, luck, chance, or external circumstances that are beyond their control, which may lead them to exercise less caution or take fewer safety measures to avoid adverse life outcomes. The concept of LoC in relation to traffic behaviour has garnered significant attention, particularly emphasizing the importance of understanding traffic LoC dynamics. Several noteworthy studies have contributed to this area of research (e.g., [41–43]. A number of empirical studies on the relationship between traffic LoC and behaviour on the roads largely focused on drivers [44–46]. A positive relationship was reported in a sample of ninety-five undergraduate students between internal LoC and positive behavioural tendencies on the roads [47]. Similarly in a recent study, while internal LoC was negatively associated with drunk driving, external LoC was positively associated with speeding and drunk driving [48]. Among a sample of Chinese drivers, it was found that external locus of control specifically on factors such as actions of other drivers was negatively associated with positive driving behaviour and vehicle/environment had a positive relationship with acceptable driving behaviour [49].

Demographic factors such as age, sex, socio-economic status, and level of education have been investigated in relation to their contribution to attitudes towards road safety and behaviour among diverse road users. While age, education and socio-economic status had no correlation with behaviour on the roads [11, 50]. Attitude towards road safety is affected by the sex of the road user [51]. Males are reported to have negative attitude towards road safety [17]. Specifically, the dynamics show that while men are involved in road traffic accidents due to violation of traffic laws, women are due to judgement errors [52–54]. It appears gender differences in driving behaviour, such as errors in inattentive driving, and driving violation remain mixed across different settings (see [17]). It is well documented in both empirical studies and accident databases that younger and novice drivers have a significant burden of road traffic accidents compared to other age groups [10, 55, 56].

### The present study

Despite the availability of studies on traffic LoC, health beliefs, risk perception, road safety attitudes and behaviour, a larger portion of those studies focused on drivers whereas little is known about motorcycle riders. It is important to note that the behaviour of drivers and motorcycle riders varies a lot among several dimensions and in the case of accidents, the survival rate also differs. Moreover, whereas the use of cars, trains, and subways are the popular means of transport in developed countries, motorcycles and tricycles are the most popular means of transport in developing countries [57] such as Ghana, yet there is little research on this dimension. Hence, the present study aimed at examining the contribution of intention to engage in health-promoting behaviour, and traffic LoC on attitudes towards road safety and behaviour in a developing country such as Ghana. Based on previous literature we hypothesized that intention to engage in health-promoting behaviour would be positively associated with more positive road safety attitudes and behaviour. External LoC would predict worse attitudes towards road safety and behaviour. Finally, we also expect that demographic factors such as age, and level of education would have a greater impact on attitudes towards road safety and behaviour.

## Method

### Participants

To determine the sample size, we considered the sample sizes reported in previous literature and a power analysis was also carried out using the G-Power software [58–60]. Considering the proposed statistical tools for the data analysis such as bivariate correlations, ANOVAs, multiple linear regressions, with a power of 90%, two-tailed tests and medium effect sizes, the minimum required sample size obtained was 269. Three hundred and seventeen (317) motorcycle riders participated in the study. However, due to incomplete data responses, the final sample size processed for data analysis was 295 (male = 76.87%). The mean age of the respondents was 27.79 (*SD* = 7.64). The study was conducted in compliance with the Helsinki Declaration and was approved by the Department of Social and Behavioural Change, University for Development Studies research ethics committee (with approval number S-2/23). The participants also signed written consent forms before completing the questionnaires. The demographic characteristics of the respondents are presented in Table 1.

**Table 1:** Demographic characteristics of the participants.

Variable	Frequency	Percentage
*Sex*		
Female	68	23.13
Male	226	76.87
*Level of Education*		
No formal education	30	10.20
Primary	11	3.74
Junior High	25	8.50
Senior High	65	22.11
College of Education	59	20.07
University	104	35.37
*Marital Status*		
Married	122	41.36
Separated	2	0.68
Single	167	56.61
Cohabitating	4	1.36
*Number of dependents*		
0	88	29.83
1	28	9.49
2	62	21.02
3	46	15.59
4	38	12.88
5	18	6.10
Above 5	15	5.09

### Study design

A cross-sectional survey design was adopted for the study. This involved presenting a set of questionnaires to the participants at once seeking their responses on a wide range of variables related to the study.

### Measures

A number of standardized scales were adopted to measure the various variables in this study. To assess riding behaviour, the motorcycle riders’ behaviour questionnaire (MBRQ: [61]) was used. Participants were asked to rate the extent to which they agree to the statements (e.g., “Not notice someone stepping out from behind a parked vehicle until it is nearly too late”) describing their behaviour that could be considered risky based on a six-point Likert scale starting from ‘‘never” to ‘‘nearly all the time”. The scale demonstrated a strong reliability outcome (Cronbach’s α = 0.92).

Attitude towards road traffic safety was measured using the road safety attitudes questionnaire [15]. The questionnaire is comprised of 13 items (e.g., “If you are a good rider it is acceptable to ride a little faster”) in Likert format with five responses ranging from strongly disagree to strongly agree. The scale demonstrated a strong reliability (Cronbach’s α = 0.9).

To assess the factors that influence the intention to engage in health-promoting behaviour such as wearing a motorcycle helmet, the Health Belief Model on wearing a helmet was adopted [62]. The scale contained 20 items comprised of subsections such as helmet-wearing behaviour and intention (e.g., “In the previous week, I always wore a helmet (100%) when riding, and/or sitting on the back of a motorcycle”), health motivation (e.g., “I mostly give importance to safety when riding a motorcycle”), perceived susceptibility (e.g., “I do not ride a motorcycle at high speed so I need not wear a helmet”), perceived severity (e.g., “If an accident happens when I am riding a motorcycle without wearing a helmet, it may cause my death”), perceived benefits (e.g., “Wearing a helmet when riding a motorcycle helps me feel safer”), perceived barriers (e.g., “I think that helmets are too expensive for their real value or benefits”), and cue to action (e.g., “I have seen advertisements on television, signs, or posters about the importance of wearing a helmet when riding a motorcycle”). The scale showed a strong reliability outcome (Cronbach’s α = 0.82).

Traffic LoC was assessed using the traffic LoC scale (Măirean et al., 2017). The questionnaire is made up of Likert-format statements reflecting various dimensions of LoC where the respondents indicate the extent to which they disagree or agree, with ratings being 1 to 6. We specifically used four subscales which include desirability (e.g., I have never broken the traffic rules), other drivers (e.g., Road accidents could be avoided especially if the other road users behaved safer), internality (e.g., My own risky driving behaviour could cause an accident), and vehicle environment (e.g., The underdeveloped road infrastructure is an important cause of traffic accidents). The reliability test showed a strong outcome (Cronbach’s α = 0.9).

To assess the traffic risk perception among motorcycle riders, the traffic risk perception questionnaire adapted from previous studies [15, 63] was used. This is a 15-item questionnaire made up of two subsections: collision event risk perception (e.g., Please indicate the likelihood of each of the following incidents leading to serious or fatal injury. Head on collision?”) and general traffic risk perception (Please indicate what you think is the general likelihood of a person being seriously or fatally injured when using the road system, As a pedestrian?”). The reliability test on the scale showed a strong outcome (Cronbach’s α = 0.96).

### Procedure

The questionnaire was hosted on Google form and trained research assistants embarked on an in-person data collection exercise, approaching motorcycle riders on major town roads and other public places. In the cases where participants could read and understand, the research assistants were expected to either hand over the tablet for self-entry or where applicable share the link to respondents personally via phone for self-completion of the questionnaire. The data collection exercise took place from the 1^st^ to the 30^th^ of August, 2023.

### Data processing and analysis

Data was cleaned using Microsoft Excel and the analysis was carried out using JASP version 0.17.2.1 [64]. For each participant, a total score was obtained for each scale by summing all the responses. Means and standard deviations were computed for all the scales which were used as the outcome variables. A test for normality showed that the data did not violate normal distribution hence parametric tests were adopted for further analysis. To explore the relationship among the outcome variables, the Pearson Product Moment Correlation was used. Multiple linear regression was used to explore the contribution of the independent variables on road safety behaviour. Finally, independent t-tests and ANOVA were used to test demographic factors on motorcycle riders’ behaviour. These specific statistical tests used in the present study find support in recent studies investigating psychosocial variables on road user behaviour [11, 55, 65, 66].

## Results

The descriptive statistics for the various outcome measures are shown in Table 2. These include the motorcycle riders behaviour (*M* = 125.49, *SD* = 22.36), traffic LoC (*M* = 116.87, *SD* = 17.76), road safety attitude (*M* = 33.72, *SD* = 10.89), risk perception (*M* = 46.12, *SD* = 15.10), and health belief model (*M* = 100.14, *SD* = 12.77).

**Table 2:** Means and standard deviation of the study variables.

Scale	sub-scale	Mean	Std. Deviation	Minimum	Maximum
Riders’ behaviour		125.493	22.363	66	187
	Traffic Errors	29.738	9.903	13	53
	Control Errors	16.905	5.873	7	31
	Speed Violations	21.126	7.817	9	45
	Stunts	13.633	5.7	7	30
	Safety Equipment	38.915	7.469	13	48
Traffic LoC		116.874	17.762	45	156
	Desirability	35.82	8.417	14	54
	Other Drivers	28.769	5.435	7	36
	Internality	23.31	5.336	5	30
	Vehicle Environment	28.976	4.57	10	36
Health Belief Model		100.14	12.77	51	137
	Helmet Intention	9.663	3.286	2	14
	Health Motive	18.065	3.047	4	21
	Perceived Susceptibility	10.173	4.83	3	21
	Perceived Severity	17.422	3.79	3	21
	Perceived Benefits	17.405	3.381	3	21
	Perceived Barriers	11.065	4.45	3	21
	Cues to Action	16.35	3.278	3	21
Road Safety Attitude		33.721	10.892	13	62
Risk perception		46.122	15.095	15	72
	Collision Risk	25.133	8.675	8	40
	General Traffic Risk	20.99	7.358	7	35

The results of the Pearson Product Moment correlation showed a significant positive correlation between attitude towards road safety and road user behaviour (*r* (295) = .33, *p* < .001) which meant that having a more positive attitude towards road safety is associated with positive behaviour on the roads. Lower LoC (which means drifting towards the internal locus of control) was associated with positive road user behaviour (*r* (295) = -.23, *p* < .001). On the association between the health belief model and road safety attitude, the various factors showed the greater the intention to use a helmet, the higher the positive attitude towards road safety (*r* (295) = .231, *p* < .001). Health motivation (*r* (295) = .404, *p* < .001), perceived benefits of wearing a helmet (*r* (295) = .447, *p* < .001), and cues to action (*r* (295) = .186, *p* < .01) were all positively associated to road safety attitude. On the other hand, perceived susceptibility (*r* (295) = -.415, *p* < .001), and perceived barriers (*r* (295) = -.416, *p* < .001) were negatively associated with road safety attitude. These patterns of associations are similar to road user behaviour as illustrated in Table 3.

**Table 3:** Means, standard deviations, and bivariate correlations among the study variables.

Variable	1	2	3	4	5	6	7	8	9	10
1. Road user behaviour	—									
2. Road Safety Attitude	0.33***	—								
3. LoC	-0.226***	-0.331***	—							
4. Risk perception	-0.037	-0.129*	0.061	—						
5. Helmet-use Intention	0.253***	0.231***	0.382***	0.008	—					
6. Health Motivation	0.177**	0.404***	0.455***	0.19**	0.327***	—				
7. Perceived Susceptibility	-0.174**	-0.415***	-0.105	-0.064	-0.158**	-0.167**	—			
8. Perceived Severity	0.098	0.29***	0.333***	0.083	0.315***	0.484***	-0.152**	—		
9. Perceived Benefits	0.13*	0.447***	0.43***	0.124*	0.373***	0.571***	-0.157**	0.542***	—	
10. Perceived Barriers	-0.261***	-0.416***	-0.104	-0.06	-0.257***	-0.102	0.516***	-0.091	-0.141*	—
11. Cues to Action	0.118*	0.186**	0.402***	-0.009	0.482***	0.276***	-0.169**	0.272***	0.309***	-0.145*

Note:

*** *p* < .001

** *p* < .01

* *p* < .05

The contribution of traffic LoC, road safety attitude, and risk perception on road user behaviour showed the model prediction as statistically significant [*F*(3, 293) = 13.73, *p* < .001] and accounted for about 35.3% of the variance on road user behaviour (*R*^2^ = .124, *R*^2^ adj = .115). The specific contribution of the individual predictors showed that traffic LoC (β = -.131, *p =* .025), and road safety attitude (β = .120, *p* < .001) significantly contributed to the model, except risk perception (β = .008, *p* = .878). The summary of the regression results is illustrated in Table 4.

**Table 4:** Summary of the results of multiple linear regression on road user behaviour.

Variable		*B*	*SE B*	ß	*t*	*p*
Model (LoC, RSA and RP)						
LoC		-0.165	0.073	-0.131	-2.255	.025
RSA		0.591	0.120	0.288	4.909	< .001
RP		0.013	0.082	0.008	0.153	0.878
R	0.353					
R^2^	0.124					
Adj. R^2^	0.115					
F	13.727					< .001

Note: *B* = unstandardized coefficient, *SE* = Standard error, ß = Standardized coefficient, LoC = locus of control, RSA = road safety attitude, RP = risk perception

We further examined individual differences based on the demographic characteristics on attitudes towards road safety and road user behaviour. The results showed that there were no significant differences between males and females on attitude towards road safety (*t*_(292)_ = 0.95, *p* = .34), and road user behaviour (*t*_(292)_ = 1.204, *p* = .23). Level of education had no significant effect on road safety attitude (*F*_(5, 288)_ = 1.196, *p* = .31), and road user behaviour (*F*_(5, 288)_ = 1.898, *p* = .095). It was further observed that age had a significant negative correlation with road safety attitude (*r* (295) = -.118, *p* < .01) but not road user behaviour (*r* (295) = -.036, *p* = .54). As the majority of the respondents (97%) were either married or single, we examined if marital status would have an influence on the attitude towards road safety and behaviour. The results showed that marital status had no significant impact on attitude towards road safety (*t*_(286)_ = 0.292, *p* = .77), and road user behaviour (*t*_(286)_ = -1.294, *p* = .197). The number of dependents had no relationship with attitude towards road safety (*r* (295) = -.04, *p* = .496), and behaviour (*r* (295) = 0.089, *p* = .13).

## Discussion

The present study examined the role of traffic LoC and health belief on attitude towards road safety and behaviour of motorcycle riders in a lower- and middle-income country. The main findings showed that having a positive attitude towards road safety was associated with increased acceptable behavioural tendencies on the roads. This is consistent with previous studies reporting a positive association between road safety attitude and behaviour among drivers [11, 14, 15]. This further implies that the relationship between attitudes and behaviour on the roads could be generalized to all types of vehicles irrespective of the level of development in a country. This explanation finds support in a previous study as well [17]. In the context of road safety, a favourable attitude can be seen as an indication that individuals perceive the advantages of adhering to safety measures to outweigh any inconvenience or effort required.

Disposition towards internal LoC was significantly associated with responsible behaviour on the roads. This meant that riders who were inclined to have control over their behaviour and have less reliance on others were more likely to be careful on the roads and adhere to traffic regulations. Hence our second hypothesis that higher LoC will be negatively associated with road safety attitudes and behaviour was partly supported. This finding is consistent with several other studies involving other road users [44, 47, 67–69]. This implies that motorcycle riders who believe in their capacity to control their own destiny and safety on the road are more inclined to adopt safer practices. This finding underscores the importance of individual empowerment and self-efficacy in the realm of road safety among motorcycle riders. Indeed, a previous study showed that when drivers are engaged in training sessions on increasing defensive driving, and safer driving practices, their internal LoC increases [45].

The intention to wear helmets based on the Health Belief Model and how that is associated with rider’’ behaviour showed varied outcomes. Specifically, the intention to wear helmets, perceived benefits, cues to action, and higher health motivation were associated with having a positive attitude towards road safety and behaviour. This partly supports the second hypothesis that intention to engage in health-promoting behaviour would be positively associated with positive behaviour on the roads. This is consistent with previous studies reporting similar patterns [32–34]. Again, the present finding that perceived barriers are negatively associated with attitudes towards road safety is supported in the literature (e.g., [38]). This suggests that the Health Belief Model may provide valuable insights into road safety-related intentions but might not fully capture the complexities of rider behaviour. Other contextual or situational factors could be at play, which necessitates further investigation.

On the influence of socio-demographic factors on attitudes towards road safety and behaviour, the results revealed intriguing patterns. Notably, we observed that sex differences did not have a significant impact on road safety attitudes and behaviours among motorcycle riders. This suggests that, in our specific context, both male and female riders hold similar attitudes and exhibit comparable behaviours concerning road safety. On the other hand, the study found that levels of education and age did not significantly influence road safety attitudes and behaviours. This is inconsistent with previous studies suggesting females exhibit more positive attitudes and behavioural tendencies on the roads [67]. This meant that our third hypothesis was not supported. These results challenge common assumptions about the impact of education and age on road safety perceptions and actions. However, they also highlight the need for further research to explore the specific cultural and contextual factors at play in this lower- and middle-income country that may influence these relationships.

The present findings have important implications for theory and practice. These findings are important for policy guidelines, especially at a time when LMICs are recording higher levels of road fatalities with limited resources to improve road infrastructure. This means that efforts aimed at reducing road carnage among motorcycle riders should pay attention to attitudinal change interventions, their health beliefs, and traffic LoC. The present findings showed the beneficial association of road safety attitude and riding behaviour which ultimately would help reduce road carnages. If motorcycle riders drift more towards the mindset that they have control over their behaviour including when riding a motorbike, they are more likely to ride cautiously and take more responsibility when on the road.

The present study addresses a critical issue in public health and safety by delving into the largely overlooked realm of motorcycle riders’ attitudes and behaviours in the context of road traffic accidents. Despite ongoing interventions, the escalating fatalities among motorcycle riders in lower-to-middle-income countries underscore the need for a more nuanced understanding of contributing factors. This study brings forth a novel perspective by examining the influence of traffic LoC and health beliefs on road safety attitudes and behaviours among motorcycle riders. Aside from traffic LoC, the incorporation of the Health Belief Model introduces a comprehensive analysis, identifying specific factors such as intention to use helmets, health motivation, perceived susceptibility, perceived benefits, and perceived barriers. These factors emerge as critical contributors to road safety attitude among motorcycle riders, offering a holistic understanding of the relationship between health beliefs and road user behaviour. For the first time, the present study has extended the literature on the role of traffic LoC and health belief on attitudes towards road safety and behaviour among the population of motorcycle riders. Whereas previous studies focused on the attitudes and behaviour of drivers, the present study examined that of motorcycle riders. Motorcycle riders appear distinct due to the heightened risk and limited protection they possess when riding their motorcycles.

## Conclusion

The present study has shed light on the intricate web of factors that shape road safety attitudes and behaviours among motorcycle riders in lower- and middle-income countries. Specifically, we found that as both road safety attitude and behaviour are positively related, internal LoC and attitude towards road safety were significant predictors of behaviour on the roads. Some factors in the Health Belief Model (i.e., intention to use helmets, health motivation, perceived benefits, and perceived barriers) were related to attitudes towards road safety. While the Health Belief Model and traffic LoC provide valuable frameworks for understanding these dynamics, they are not exhaustive, and the influence of other individual, cultural, and contextual factors must be considered. Moreover, the findings emphasize the importance of fostering positive attitudes, enhancing self-efficacy, and tailoring road safety interventions to the unique characteristics of the population considering the inadequate resources to comprehensively deal with the road carnages. Further research is needed to explore the complex interplay of these factors and to develop more effective strategies for promoting road safety in this context.
